# The Outcome-Present State Test Model of Clinical Reasoning to Promote Critical Thinking in Psychiatric Nursing Practice among Nursing Students: A Mixed Research Study

**DOI:** 10.3390/healthcare11040545

**Published:** 2023-02-12

**Authors:** Yu-Chin Ma, Jin-Ling Jiang, Yu-Chuan Lin

**Affiliations:** Department of Nursing, Tzu Chi University, 701 Zhongyang Road, Section 3, Hualien 97004, Taiwan

**Keywords:** critical thinking, mixed study, outcome-present state test model

## Abstract

This study determined whether teaching intervention using the outcome-present state test (OPT) clinical reasoning model can effectively improve critical thinking in nursing students during a psychiatry internship. In addition, it evaluates the experiences of the students using this model in clinical practice. Methods: In this interventional study, 19 students were taught critical thinking skills using the OPT clinical reasoning model during a psychiatry clinical practice. Work-learning forms were used in daily 1 h individual and group discussions with students. The critical thinking disposition scale was completed by every student before and after the intervention. Moreover, the students were asked to the complete reflection experience forms. Results: The average critical thinking disposition pre-intervention score was 95.21, whereas the average post-intervention score was 97.05, indicating an increase of 1.84. There was a significant increase in the fourth dimension of open-mindedness (z = −2.80, *p* < 0.01). The learning experience has been likened to a process of clearing the fog, and it involves the use of limited known conditions, thinking outside the box, and adaptation to complex care issues. Conclusion: Using the OPT clinical reasoning model as a teaching strategy during a psychiatric nursing internship significantly improved the open-mindedness dimension among the students. The student reflective experience of talking to teachers as peers helped students identify clues and reframe problems related to clinical care. Additionally, the students reported that this led to more harmonious interactions with their teachers.

## 1. Introduction

Clinical practice is a core module of nursing education. During this practice, students acquire knowledge and learn practical skills in the clinical field. They also have the opportunity to apply what they have learned. In clinical practice, the role of the teacher is to optimize students’ learning. Thus, the teachers must find ways to promote proactive learning among their students. Notably, the critical thinking skills of nurses can affect care outcomes. Therefore, cultivating critical thinking skills in nursing students and integrating them into nursing education is necessary for students to gain sufficient professional responsibility [1].

Critical thinking is a complex activity, and its development requires education, time, and personal commitment [2]. The teachers’ ability to assess students is important for measuring critical thinking skills and monitoring their development [3]. Clinical reasoning as an analytical process used to solve clinical problems and patient care [4]. Although many explanations have been reported in the literature, there is a consensus that critical thinking is oriented thinking involving reflective reasoning before having a conclusion that leads to a clinical decision. Critical thinking is the intellectually disciplined process of actively and skillfully conceptualizing, applying, analyzing, synthesizing, and evaluating information [5]. The review of tools to measure critical thinking in nursing and midwifery students noted that the measurement of critical thinking in some studies reviewed may have been influenced by the impact of culture on different learning environments [6]. Of the 53 papers that were reviewed, the majority (n = 38) measured critical thinking. The other 48 papers focused on describing and/or validating a measurement tool or model. Multiple tools were used to measure critical thinking. Of the commercially developed tools, the most common were the California Critical Thinking Skills Test (CCTST) and California Critical Thinking Disposition Inventory (CCTDI) and variations of CCTST and CCTDI. The CCTST and the CCTDI were the only commercially developed tools that were translated into other languages [7].

Critical thinking is of great importance, but studies have showed that nursing students lack critical thinking dispositions and skills [8]. The improvement in critical thinking skills among nursing students can positively affect self-reflection and care behavior [9]. Researchers suggested that educational interventions including use of explicit teaching strategies and higher-order questions are to be promoted in advancing students’ critical thinking [10,11]. In the hospital, nurses are required to make important and quick decisions that have consequences on the health of their patients. Therefore, quick thinking and sufficient foresight to accurately predict the possible outcomes of intervention choices are central aspects of nursing. Thus, critical thinking skills need to be integrated in university nursing education. In addition, teaching methods and strategies should be changed to include reflection and development of clinical judgment for effective and ethical problem solving [12].

The Singapura study was to explore nursing students’ perceptions of critical thinking and clinical reasoning. The nursing students in this study felt that critical thinking and clinical reasoning were essential skills for providing high-quality patient care [13]. In nursing education, critical thinking is an essential skill for clinical reasoning and optimal nursing practice to improve patient care, and, thus, has become one of the most highly valued educational outcomes recently [14]. The study findings showed that nursing instructors must implement teaching strategies to develop critical thinking among students [15]. These teaching strategies should incorporate critical thinking into overall learning to encourage its use in clinical practice [16,17].

Pesut and Herman (1998) proposed the outcome-present state test (OPT) model to provide students with clinical reasoning tools to be used in complex scenarios [18]. The OPT model maps the relationships between the patient’s disease diagnosis and the associated care issues. This map is created by summarizing the patient’s background information and incorporating the knowledge of the nurses. A previous study reported that the clinical reasoning web can visualize the relationship between patients’ diagnoses and their care needs, allowing students to prioritize care problems as well as determine dynamic, interactive, and causal relationships between the contributing variables [19]. The study found that the use of the clinical reasoning web concept map resulted in significantly better problem solving, support, and understanding among nursing students. The web concept map to improve critical thinking in nursing students [20].

Research has shown that the critical thinking disposition is positively associated with effective clinical decision-making and competence among the clinical nurses [21], which subsequently improves patient safety and enhances patient outcomes [22]. Our study was conducted with nursing students through the learning strategies using the self-regulated learning (SRL) model and the OPT model of reflective clinical reasoning. SRL is a model of learning situated in social cognitive theory that views learners as active participants in their learning. The current study determines whether the OPT model can effectively improve critical thinking in nursing students during a psychiatry nursing practice and to understand students’ reflection experiences of using this clinical reasoning model.

## 2. Methods

### 2.1. Study Design

In this interventional study, 19 nursing students were taught to use the OPT model during a psychiatry nursing practice. Signed consent forms were obtained from all participants. The critical thinking disposition scale was completed by the nursing students before and after the practice.

### 2.2. Subjects and Setting

Overall, 19 third-year students from a nursing university participated in this study. Participants were also required to complete a psychiatric nursing practice. The practice was in the Tzu Chi Hospital psychiatry ward. These participants were classified into three groups of 5–7 students. The practice duration was 3 weeks. The practice instructor was also the researcher in this study.

### 2.3. Intervention

During the first week, in order to think about the patient problem, we used a clinical reasoning web worksheet. A group discussion was then conducted in which the students shared the contents of their worksheets and discussed the background of their patients. Students were encouraged to help each other identify clues, such as the patient’s major complaints, test results, and symptoms, and to use these to illustrate what they had learned about the patient’s history. They were also encouraged to help other to present their patient’s diagnosis.

In the second week, we used the clinical reasoning web worksheet, including a concept map, to help students think about the following: 1. Current evidence, 2. The relationships between the evidence and the nursing diagnosis, 3. How the nursing diagnosis was reached and how important it was, and 4. A topic summarizing the patient’s background history.

In the third week, the teacher and students completed the clinical reasoning web worksheet together. During the group discussion, the teacher encouraged the students to think about the following: 1. The significance of the tests being administered (current/pending results), 2. How the patient defined their current condition, 3. The gap between the patient’s expected outcomes and their current condition; 4. How these gaps might be filled, and 5. Whether thoughts about care measures or clue symptoms should be reviewed.

In addition, the students completed a reflection log as well as worksheets. A human pictogram was used to assist the students in completing the patients’ clinical care reports.

### 2.4. Data Collection

#### 2.4.1. Critical Thinking Disposition Scale

The Critical Thinking Scale is a self-development questionnaire by Yeh (1999) and was used to measure the critical thinking disposition of students. This scale is a 20-item, six-point Likert-type scale (ranging from 1 = No to 5 = Always). The Critical Thinking Scale was four dimensions: systematicity and analytical skills (9 items), open-mindedness and empathy (4 items), knowledge and curiosity (3 items), and overall reflection (4 items). Scores from the 20 items can be added up to obtain a total score, ranging from 20 to 120. The Cronbach’s alpha of the overall scale is 0.86 and subscale α values ranging from 0.53 to 0.76 [23].

#### 2.4.2. Reflection

Reflection homework was completed by the participants, and the students were required to answer three questions.

1. What was the most striking thing about the patient that you cared for in this internship and why?
2. During the internship period, how has the clinical reasoning web and OPT clinical reasoning model worksheet helped you/your patient?
3. What difficulties did you encounter when using the clinical reasoning web and OPT clinical reasoning model worksheet during the internship?

### 2.5. Data Analysis

#### 2.5.1. Quantitative Data

SPSS Statistics for Windows version 22.0 (IBM Corp. Armonk, NY, USA) was used for the statistical analysis of data. Parametric data were presented as mean and standard deviation, whereas nonparametric data were presented as median and percentage. Wilcoxon tests were used to identify pre- to post-intervention changes in critical thinking.

#### 2.5.2. Qualitative Analysis

The students’ learning experiences were organized, and the data analysis was started with the transcription of the interview recordings and the preliminary organization of the transcripts. The investigator carefully read the transcripts as well as extracted descriptions of clinical scenarios and the reasoning used to form meaningful units. Units with similar contents were grouped into themes, and themes with similar concepts were grouped into categories. Furthermore, the investigator repeatedly reviewed the transcript data to increase the depth and breadth of the categories. The rigor of the data was trustworthiness, credibility, transferability, and conformability [24]. During the interviews, the investigator adopted a sincere nonjudgmental attitude to encourage the respondent to freely disclose his/her thoughts. The content of the interview was faithfully transcribed, and respondents were encouraged to describe their experiences in as much detail as possible. The students were considered coinvestigators, and the results were sent to the student for confirmation before the study was completed. In addition, the investigator described his/her own experiences, background, and thoughts to ensure that the entire study process was documented in detail. The researchers with peer discussions were repeated during the data analysis. Throughout the study, the researcher continued to study the relevant literature, reflecting on his/her experiences, and discussing his/her perspectives.

### 2.6. Ethical Considerations

This study was approved by the Institutional Review Board of Tzu Chi Hospital (IRB-110-158-B) and was conducted in accordance with the principles of the 2013 revision of the Declaration of Helsinki. Participants were invited to participate in the OPT clinical reasoning model and informed that their learning rights would not be compromised if they left the study early. All participants signed an informed consent form.

## 3. Results

### 3.1. Demographics

The study included 19 participants; of these, 13 (69%) were women and 6 (31%) were men. The mean age of the participants was 21.4 years, and the mean course satisfaction score was 4.99.

### 3.2. Quantitative Research Results

As shown in Table 1, the average pre-investigation critical thinking disposition score of the 19 students was 95.21, and the average post-investigation score was 97.05, indicating an increase of 1.84. Among the four critical thinking disposition dimensions, the post-investigation score for systematicity and analytical skills decreased by 1.21 from the pre-investigation score, but the difference was not significant. The score for open-mindedness had increased by 1.39 post-investigation, and the difference was significant (z = −2.80, *p* < 0.01). Additionally, the scores for curiosity and overall reflection increased by 0.9 and 0.73, respectively, and there were no differences between the pre- and post-investigation scores.

**Figure 1:**
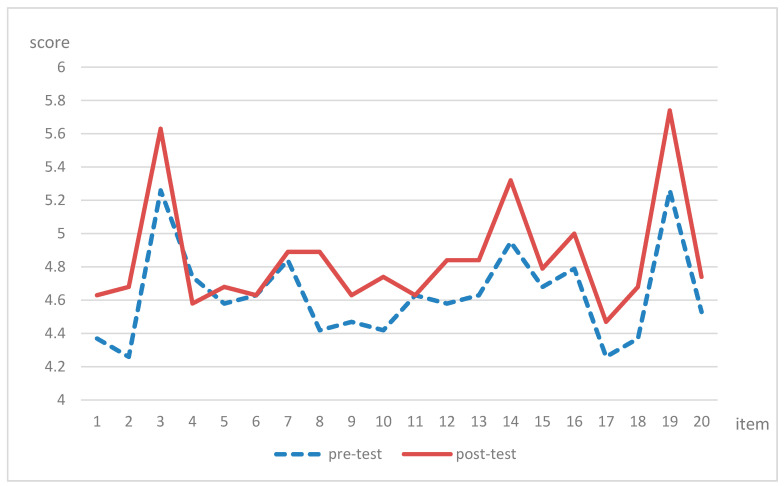
The average score for each item in the critical thinking scale (n = 19).

As shown in Figure 1, the worst scoring items on the critical thinking pre-test were item 2 “I attempted to use some new viewpoints or concepts,” and item 17 “When others proposed a viewpoint, I attempted to identify the implicit main arguments in the viewpoint.” The critical thinking scale item 8 that showed the maximum improvement was “When handling problems, I first define the problem clearly,” and the average score increased from 4.42 to 4.89.

### 3.3. Qualitative Research Results

Nursing students are unfamiliar with clinical care scenarios during their internship, are unaware of what to do, and may feel scared and anxious. This learning process is like entering a fog (Table 2). Using appropriate teaching strategies, the internship instructor can guide students in clinical reasoning discussions and teach them to make multiple connections using limited information and identify their focus of care.

#### 3.3.1. Using Limited Known Conditions

The internship process is like solving a puzzle. Students learn to gather information about the clinical care of the case such as the patient’s chief complaint or test results and try making several connections. Therefore, any piece of information may pose a nursing issue that could be overlooked if neglected.

##### Identifying Problems from Scraps of Scattered Information

I had to identify important information based on scraps of information collected from the patient and collate it into the reasoning web and OPT worksheet. Problems could have occurred if the wrong key points were extracted during this transformation process. (B3).

I feel that there is a need to link the patient’s symptoms with lifestyle habits before admission or at initial disease onset. I had to frequently ask the patient about his/her medical history or family and work information again. (C3)

I am unclear about the integration of observation data and symptoms, and this may affect subsequent nursing problems and measures. There will be omissions in nursing problems if the observation data are extremely few. (K3)

##### Multidimensional Connections

I should try making multidimensional connections. Every piece of information may be a nursing problem, and omission of this will lead to overlooking the problem. If the problem is not resolved, the objective of this hospitalization will be lost. (E3)

I feel that using the OPT worksheet can allow me to better connect the patient’s problems and find correlations between the problems, which allows me to reflect on which management measures are better. Additionally, I would think of any difficulties the patient is facing, such as, measuring blood sugar at home, and then think about other methods to solve this problem. (L2)

#### 3.3.2. Thinking outside the Box

Students used several problems and clues to learn how to clarify the context of a problem. During this process, the teacher and students worked together to identify different viewpoints and problem causality.

##### Clarifying the Context of the Problem

I can better analyze and understand the details omitted by me, more comprehensively think about problems, and identify blind spots and overcome them. Discussion with the teacher allowed me to comprehensively analyze nursing problems. (F2)

I feel that the reasoning web and OPT worksheet can help me better clarify the context of the problem. Simultaneously, I can better confirm nursing problems that match the patient’s condition and decrease problems that lead to misjudgment errors and increased length of hospitalization. In addition, this method allows for efficient and effective provision of nursing measures. (D2)

##### Adjusting One’s Thoughts

The main part of this theme is to adjust one’s own thoughts. This is because the information received is sometimes scattered and cannot be networked, resulting in an inability to use it for the patient. When the teacher asked questions, I kept repeating and going in circles and was unable to rearrange the patient care framework and derive new care interventions. (G2)

The main assistance is that it organizes thoughts, identifies primary and secondary problems, and allows me to think about the ways to provide suitable treatment. (J2)

#### 3.3.3. Adapting to Complex Care Issues

Students became adept at using the OPT worksheet and implementing the strategies taught. After learning to adjust their thinking, they were better able to understand their patients’ clinical care problems and made fewer care omissions. The reframing proposed by the students was difficulties in learning.

##### Accurate Care Focus

I feel that these two reasoning tools allow me to clearly understand the priority and causality of the patient’s problems such that I could efficiently and accurately understand the focus of care, thereby determining the correct nursing problems and measures. (A2)

I feel that the greatest assistance rendered is in recognizing the patient’s current symptoms, and there is a clear care focus on implementation. (H2)

##### Decreasing Care Omissions

This form can help determine my patient’s current condition and problems and to examine them individually by analyzing various aspects. The form also allows me to know what I have omitted in addition to identifying the main issues. (E2)

##### Reframe Learning

I feel that this is a reframing, and it is difficult to understand as I need to consider the patient’s difficulties before thinking of other suitable methods. However, I became enlightened after discussing it with my teacher. (L3)

### 3.4. Compilation of Qualitative and Quantitative Results

The critical thinking scores were increased. This indicates that the OPT model teaching strategy helped improve critical thinking among nursing students. The students also reported that the learning process improved collaborative learning and their interactions with teachers. The students’ reflections on their experiences revealed that thinking outside the box and adjusting thoughts to become more problem-focused were effective. This result is consistent with the significant improvements in open-mindedness observed using the critical thinking disposition test.

In addition, nursing students reported that they could better adapt to complex nursing problems using the OPT model to quickly identify their patients’ nursing problems and that they could reduce their nursing care omissions. This explains why the students indicated that the clinical reasoning model provided a deeper understanding and improvement in their critical thinking after the internship was completed. The nursing students’ reflections on their experiences indicated that they viewed the OPT teaching method as a student-centered learning experience that required them to identify cues, think about those cues in context, connect variables within problems, and think outside the box. This learning experience inspired deeper learning and enhanced their cognitive abilities. This was demonstrated by the increase in the scores for critical thinking disposition in the quantitative results.

## 4. Discussion

Our study was conducted with nursing students through the learning strategies using the SRL model and the OPT model of reflective clinical reasoning. The SRL model in nursing is proposed as a theoretical structure that explains how clinical reasoning skills can be acquired through attention to reflective thinking and critical thinking skills. This approach aligns with nurse education’s underpinning philosophical approach to learning in fostering critical thinking and to be independent and lifelong learners [25]. Evidence suggests that effective and efficient clinical reasoning is a consequence of intentional reflection supported by self-regulation [26]. A study findings included the high level of reported motivational and learning strategies used by students in their approach to learning, and in their teachers as partner [27].

The critical thinking scores of nursing students improved after using the OPT clinical reasoning model as a teaching strategy during their psychiatric internship. This result is consistent with the result of a previous study that reported that improved critical thinking can help nursing students to more accurately use it in nursing internships and to become better at dealing with complex and challenging clinical scenarios [8]. In this study, worksheets were collaboratively completed by the teacher and students, and the students were guided to think about the connections between the clues and problems. This encouraged them to express their thoughts and learn to think contextually about their patient’s health. Nursing students perceived critical thinking and clinical reasoning as essential for nursing practices and described these skills as linking theory to practice. Strategies such as simulation, case studies, real clinical experiences, and guidance from clinical instructors/preceptors were found to stimulate critical thinking and clinical reasoning for the students. It is necessary to train and enhance smooth transition from theoretical to practical healthcare practices [13].

Reflections on the students’ experiences generally indicated that they found the OPT model worksheet helpful in accurately identifying the focus of the care situation, but they had difficulty in reframing the learning. When students were guided to think about the differences between patient-expected solutions and current care, whether the revision in care interventions was needed and whether they felt able to engage patients in discussions about their expectations, they generally found it difficult to answer such questions. Some students have proposed the challenges of critical thinking. The results of this study, which are consistent with earlier literature, show that students have difficulty in learning critical thinking processes, which can hinder the development of their self-confidence in critical thinking [28]. One explanation may be that students have been used to the Chinese conventional pedagogy that values rote-memorization and respecting authoritative figures (teachers) influenced by the Confucian culture. In the Chinese educational evaluation system that is often exam-oriented, there is less encouragement for having questions from students [29]. The study corroborates the assertion that students’ understanding and practice regarding critical thinking are shaped by social, cultural, and educational contexts [30].

The care culture in Taiwan was traditionally dominated by medical personnel, with patients expecting these professionals to effectively manage their healthcare and medical treatment. These cultural factors contribute to an individual’s critical thinking disposition [31]. The study of university students from New Zealand and Japan found that culture-related factors do influence students’ critical thinking use. However, the variations in these factors may not always correspond to differences in critical thinking. Their findings showed that there were no appreciable variations in the reported use of critical thinking between students from Western and Asian cultural backgrounds. Students’ use of critical thinking is more significantly impacted by the educational environment [32]. Therefore, nursing education in Taiwan must change. The students must learn to consider the patient’s perspective on patient outcomes and embrace patient-centered nursing practice.

The reflections of the nursing students in this present study reported that the use of the OPT worksheet helped them understand clinical nursing problems from the patient’s perspective and to reduce care omissions. Clues and concepts can be linked and discussed at different stages of the nursing process to strengthen the clinical preparation of such students. Students also learned to plan their care strategies and prioritize the most important items. This finding is consistent with previous research results indicating that critical thinking facilitates the nursing process [33]. In the study, lecturers presented several patient problems to the group and guided students to work from initial hypotheses based on prior knowledge or experience, and then collected data for further synthesis and analysis of health problem solutions and nursing intervention. The learning outcome design of the course tools were introduced to help students develop critical thinking, such as reflective journals of this course. These tools could cultivate students’ elaboration of critical thinking [14]. This methodology, such as interactive teaching strategies and student active participation, together contributed to the improvement of students’ critical thinking and creative self-efficacy [28]. Additionally, implementing a teaching strategy for critical thinking in clinical scenarios can enhance students’ theoretical and clinical competencies and skills as well as better prepare them for their future careers as professional nurses.

## 5. Conclusions

The OPT clinical reasoning model as a teaching strategy in a psychiatric nursing internship significantly improved critical thinking among the students. Teachers encouraged students to find clues and reframe clinical care problems. Students reported that they felt a sense of accomplishment and experienced more harmonious interactions with their teachers. We also found that students had difficulty understanding critical thinking as an abstract concept, but they could use worksheets and images to discuss and present concept map findings. The students found that this helped in easier understanding.

## 6. Limitations

This study has some limitations. The sample size was small, and generalizability was insufficient. Therefore, further studies on the OPT clinical reasoning model and critical thinking are needed to validate our study results.

Based on our findings, we propose two suggestions. First, we recommend that teachers introduce the OPT clinical reasoning worksheet during nursing internships and use images to facilitate discussion. This can effectively improve students’ understanding of the critical thinking process. Second, we recommend that the development and use of measure tools include care situations of critical thinking ability in future research. Although the critical thinking scale was used in this study, the questions excluded clinical scenarios that nursing students may face, leading to some discrepancy between the scale and the clinical learning content. The ward culture in a clinical setting is a significant aspect that influences how students learn to use clinical reasoning and critical thinking. The results of this study did not bring up the critical thinking discussions related to the interaction process with the nursing staff. Future research can explore the clinical reasoning learning experience of nursing students and clinical nurses during the practice process.

## Figures and Tables

**Table 1:** Critical thinking about differences in various aspects pre-post psychiatric practice (*n* = 19).

Critical Thinking	Pre M(SD)	Post M(SD)	z	p
Total	95.21 (17.01)	97.05 (10.59)	−1.42	0.155
analytical	44.47 (13.68)	43.26 (5.17)	−1.22	0.222
open-mindedness	19.63 (2.06)	21.02 (1.71)	−2.80	0.005
curiosity	13.10 (1.37)	14.00 (1.97)	−1.70	0.089
reflection	18.00 (3.12)	18.73 (2.55)	−0.59	0.155

**Table 2:** Categories and subcategories of the study.

	Categories	Subcategories
Clearing the Fog	1. Using limited known conditions	1. Identifying problems from scraps of scattered information 2. Multidimensional connections
	2. Thinking outside the box	1. Clarifying the context of the problem 2. Adjusting one’s thoughts
	3. Adapting to complex care issues	1. Accurate care focus 2. Decreasing care omissions 3. Reframe learning

## Data Availability

The data presented in this study are available on request from the corresponding author.
